# The Efficacy of Three Rotary Systems (Reciproc Blue, WaveOne Gold, and AF Blue R3) in Preparing Simulated, Highly Curved Root Canals: An In Vitro Study

**DOI:** 10.7759/cureus.30232

**Published:** 2022-10-12

**Authors:** Hadeel Al-labed, Kinda layous, Hasan Alzoubi

**Affiliations:** 1 Department of Restorative Dentistry and Endodontics, Damascus University, Damascus, SYR; 2 Department of Pediatric Dentistry, Damascus University, Damascus, SYR

**Keywords:** root canal, waveone gold, preparation time, canal centralization, simulated canals, af blue r3, reciproc blue

## Abstract

Purpose

This study aimed to evaluate the effectiveness of three rotary, single-file, and reciprocating systems in terms of preparation time and canal centralization in simulated highly curved root canal preparation.

Materials and methods

The study sample consisted of 45 simulated canals with a curvature of 40°. They were randomly distributed into three experimental groups: Group 1-Reciproc Blue; Group 2-WaveOne Gold; and Group 3-AF Blue R3. A glide path was established by #10 hand K-file, then red dye was colored on the simulated canals, and photographs of the sample were taken before preparation. Then, the canals of each group were prepared, and other photographs were taken after preparation. The images of the two phases were combined using AutoCAD, where the canal was divided into three parts and the preparation area was measured from the right and left sides of each part of the canal. The preparation time for each system was also measured.

Results

While there were no statistically significant differences in the rate of preserving canal centralization in the middle and apical thirds between preparation groups, a statistically significant difference was found in the coronal third, as the Reciproc Blue and WaveOne Gold systems have a greater ability to maintain the centrality of the canal compared to the AF Blue R3 system. While there were no statistically significant differences between the Reciproc Blue and WaveOne Gold systems in the coronal third, as for the preparation time, it was found that there were statistically significant differences in the preparation time between the groups in favor of the WaveOne Gold system.

Conclusion

Both preparation systems (Reciproc Blue and WaveOne Gold) maintained the anatomical shape and canal centrality, with more cons for WaveOne Gold compared to the Reciproc Blue system. Regarding the volumetric changes, AF Blue R3 had the greatest changes compared to the Reciproc Blue and WaveOne Gold systems. WaveOne Gold Group, in terms of canal preparation time, showed the least time among the investigated groups.

## Introduction

Mechanical preparation must maintain the shape and orientation of the initial anatomy of the canal. This ideal preparation is considered a challenge in curved canals, as root canal debridement has an important role in creating a good space for irrigation solutions, in addition to obtaining a suitable geometric shape to ensure good sealability. This must be done without changing the shape, path, or curvature of the root canal [1]. Many studies have proven that it is rare for the roots of the teeth to be completely straight, as most root canals show curvatures within different levels, and therefore it was necessary to expect this even if the radiographs failed to show these curvatures [2].

Since the root canals have many anatomical curves, the introduction of nickel-titanium alloy into endodontic therapy has allowed the production of many files of high flexibility and resistance, with a variety of shapes, tapers, and cross-sections to suit different cases [3]. In 2008, a new approach to nickel-titanium alloy was introduced in which a single reciprocating file was manufactured to reduce preparation time and cost, apical debris extrusion, and improve shaping procedures [4].

The reciprocating motion is an evolution of the balanced force technique in which the file rotates counterclockwise (CCW) by 150° [5]. This movement is followed by a 30° clockwise (CW) motion, and after completing three reciprocating rotations, the file rotates a full counterclockwise rotation [6]. Reciprocating motion has shown that the risk of file breakage due to cyclic fatigue is reduced by reciprocating motion because rotating the file counterclockwise reduces the torsional stress during canal shaping [7].

The flexibility and shape memory of the nickel-titanium files remain a source of great challenge within curved canals, as these tools left areas of the canal unprepared, and this is mainly due to the anatomical features of the root canals such as concavities, curvatures, cavities, and bifurcations, which prevent reaching the full working length and the occurrence of distortions in the shape of the canal [8].

As a result, the purpose of this study was to compare the ability of three different rotary single-file preparation systems in terms of preparation time and canal centrality in highly curvature simulated canals. 

## Materials and methods

This in vitro study compares the preparation time and canal centrality of three rotary, single-file, different cross-section, and reciprocating systems in highly curvature simulated canals (Reciproc Blue, WaveOne Gold, and AF Blue R3). The study protocol was approved by the Scientific Research and Postgraduate Board of Damascus University, Ethics Committee of Damascus University, Damascus, Syria (No. UDDS-645-25112019/SRC-3865).

**Figure 1:**
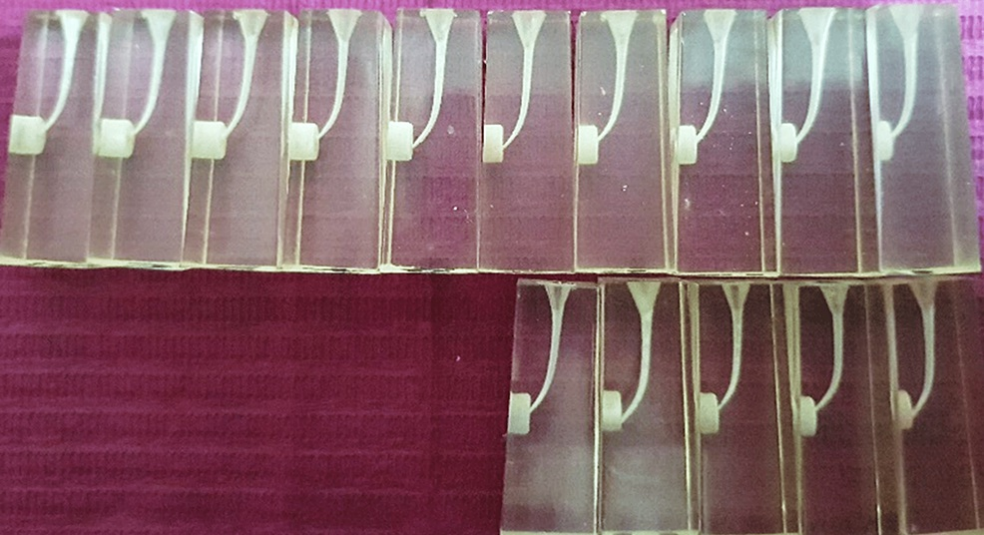
Simulated canals.

The study sample consisted of 45 canals with single apical curvature of 40° made of transparent resin. All models were similar and had unprepared canals. The diameter and taper of each of these canals were (foramen diameter 0.15 mm and taper 0.02 mm), and the working length of each canal is 16 mm, with the coronal straight segment being 12 mm and the apical curved segment being 4 mm (Figure 1).

The sample size was determined using a sample size calculation program (PS Power and Sample Size Calculation Program, Version 3.0.43). Sample size calculation produced a required sample size of 15 simulated canals per group to detect a significant difference (90% power, two-sided 5% significance level). The sample was divided into three groups; each group included 15 simulated canals according to the rotary systems: Group 1-Reciproc Blue; Group 2-WaveOne Gold; and Group 3-AF Blue R3.

A glide path was established with a #10 hand K-file, and then red dye was used to color the simulated canal. These resin models were then fixed to a base with a specific place for simulated canals and photographed using a digital camera (Canon 5D Mark IV, resolution 30.4 megapixels, Japan) at a fixed distance of 20 cm. Then the simulated canals were prepared using VDW Silver Reciproc, the silver endo motor from VDW Dental, Germany.

Group 1: The simulated canal was prepared using WaveOne Gold primary rotary files (25, 0.7) with an alternating parallelogram cross-section at a speed of 350 rpm, where the file was inserted slowly with ethylenediamine tetraacetic acid (EDTA), 19% without pressure, and the range of movement of the file was 3 mm. After every three successive movements, the file was taken out, cleaned, and washed with distilled water until the full working length was reached. Only one file was used for each canal.

Group 2: The simulated canal was prepared using a Reciproc Blue rotary file (25, 0.8) with a cross-section of an s-shaped spiral with two cutting edges and a non-working tip at a speed of 300 rpm using a silver endo motor (VDW), and the work was continued according to the previous protocol.

Group 3: The simulated canal was prepared using an AF Blue R3 rotary file (25, 0.6) with a rectangular cross-section at 300 rpm using a silver endo motor (VDW), and the work was continued according to the previous protocol.

The samples were photographed using a digital camera with the same imaging conditions before preparation so that a final image was obtained for each canal after merging the primary image, colored with red pigment, with the image after preparation. The left and right preparation areas were measured as the study was conducted at 13 levels, which were determined along the canal based on the initial image (A-B-C-D-E-F-G-H-I-J-K-L-M). These levels were equidistant from a point (M) with a difference of 1 mm, where: point M is at the end of the working length, and point L is midway between K and M (this point is set because of the importance of this region in the success of endodontic treatment).

**Figure 2:**
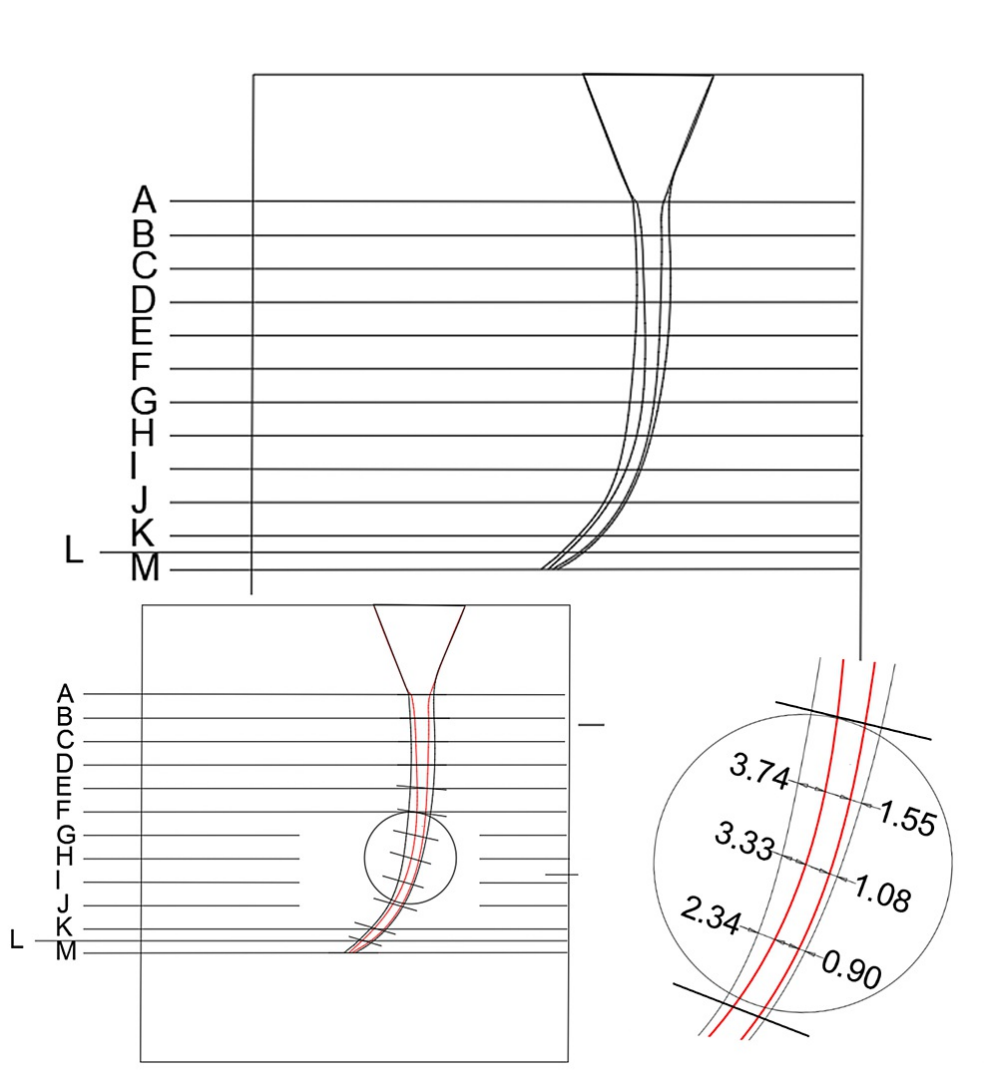
Studied levels: the canal was divided into three regions: the coronal third from the points (A-D), the middle third from the points (E-H), and the apical third from the points (I-M).

To define canal transmission, the canal was divided into three regions: the coronal third from the points (A-D), the middle third from the points (E-H), and the apical third from the points (I-M) (Figure 2).

**Figure 3:**
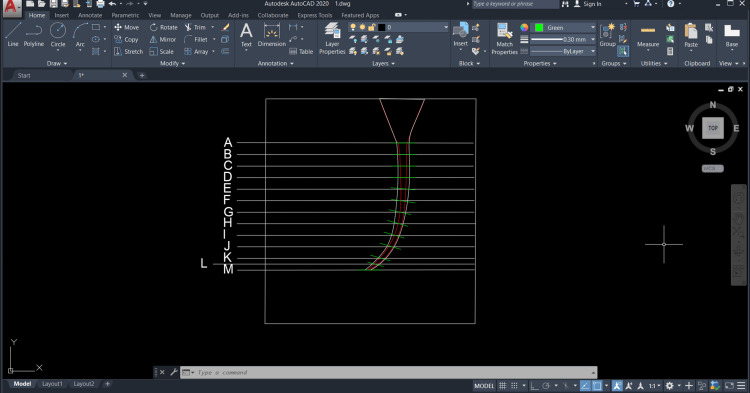
Inserting images into AutoCAD.

Work was done using AutoCAD Autodesk 2020 on the image before preparation, where the lines of the required study sections are placed on it (Figure 3). The centrality of the canal for each level was evaluated according to the following equation: the minimum value of resin removed on one side ÷ maximum value of the amount of resin removed on the other side for the same section. A value of one was taken as a criterion for maintaining canal centrality.

## Results

The study sample consisted of 585 different sections that were identified in 45 simulated root canals with 40-degree curvature. The mean values of the canal centrality preservation rate were calculated in the studied sections among the three groups (Table 1).

**Table 1:** The arithmetic mean and standard deviation of the values of the canal centralization rate in the studied points.

Sections	Preparation system	Number of measures	Mean	Standard deviation	Min	Max
Section A	Reciproc Blue	15	0.790	0.160	0.400	0.959
	WaveOne Gold	15	0.773	0.185	0.452	0.969
	AF Blue R3	15	0.612	0.112	0.405	0.767
Section B	Reciproc Blue	15	0.804	0.145	0.486	0.978
	WaveOne Gold	15	0.730	0.159	0.388	0.964
	AF Blue R3	15	0.551	0.119	0.381	0.790
Section C	Reciproc Blue	15	0.800	0.143	0.585	0.998
	WaveOne Gold	15	0.740	0.148	0.476	0.994
	AF Blue R3	15	0.582	0.154	0.372	0.834
Section D	Reciproc Blue	15	0.730	0.186	0.290	0.988
	WaveOne Gold	15	0.741	0.174	0.403	0.977
	AF Blue R3	15	0.598	0.170	0.362	0.937
Section E	Reciproc Blue	15	0.590	0.231	0.200	0.907
	WaveOne Gold	15	0.669	0.187	0.334	0.964
	AF Blue R3	15	0.579	0.204	0.318	0.935
Section F	Reciproc Blue	15	0.432	0.200	0.149	0.763
	WaveOne Gold	15	0.637	0.202	0.264	0.896
	AF Blue R3	15	0.573	0.197	0.313	0.967
Section G	Reciproc Blue	15	0.395	0.252	0.106	0.924
	WaveOne Gold	15	0.636	0.258	0.175	0.984
	AF Blue R3	15	0.583	0.220	0.202	0.976
Section H	Reciproc Blue	15	0.460	0.271	0.133	0.966
	WaveOne Gold	15	0.606	0.257	0.124	0.976
	AF Blue R3	15	0.656	0.238	0.173	0.960
Section I	Reciproc Blue	15	0.554	0.255	0.193	0.963
	WaveOne Gold	15	0.650	0.248	0.231	0.996
	AF Blue R3	15	0.629	0.238	0.292	0.961
Section J	Reciproc Blue	15	0.556	0.199	0.279	0.843
	WaveOne Gold	15	0.579	0.256	0.148	0.976
	AF Blue R3	15	0.652	0.216	0.298	0.985
Section K	Reciproc Blue	15	0.480	0.254	0.081	0.962
	WaveOne Gold	15	0.476	0.218	0.129	0.897
	AF Blue R3	15	0.428	0.162	0.226	0.746
Section L	Reciproc Blue	15	0.453	0.226	0.110	0.783
	WaveOne Gold	15	0.482	0.228	0.165	0.922
	AF Blue R3	15	0.458	0.228	0.185	0.890
Section M	Reciproc Blue	15	0.443	0.221	0.100	0.864
	WaveOne Gold	15	0.464	0.295	0.024	0.863
	AF Blue R3	15	0.418	0.201	0.018	0.870

To study the effect of the three studied systems on the rate of maintaining centrality, the one-way ANOVA (analysis of variance) test was applied (Table 2). The results show that there were no statistically significant differences in the rate of maintaining the centrality of the canal at the points (Section D, Section E, Section H, Section I, Section J, Section K, Section L, Section M).

**Table 2:** The results of one-way ANOVA tests for the studied points. * indicates .05 level of significance ** indicates .01 level of significance

Variable	Sections	ANOVA results	p-value
Canal centralization	Section A	6.008	0.005**
	Section B	12.593	0.000**
	Section C	8.677	0.001**
	Section D	3.041	0.058
	Section E	0.841	0.438
	Section F	4.134	0.023*
	Section G	4.053	0.025*
	Section H	2.366	0.106
	Section I	0.625	0.54
	Section J	0.744	0.481
	Section K	0.274	0.762
	Section L	0.071	0.932
	Section M	0.14	0.87

On the other hand, there were statistically significant differences in the rate of maintaining canal centrality in the points (Section A, Section B, Section C, Section F, and Section G), and the p-value ranged between 0.000 and 0.025. The Bonferroni test was used for the comparison of the two groups (Table 3). The results show that there was a statistically significant difference in the rate of maintaining the centrality of the canal in points (A, B, and C), where the Reciproc Blue and WaveOne Gold groups outperformed the AF Blue R3 group. It also found a statistically significant difference at Point F, where the WaveOne Gold group outperformed the Reciproc Blue group. While at Point G, Reciproc Blue and AF Blue R3 outperformed Reciproc Blue. 

**Table 3:** Bonferroni test results for comparison, maintaining the centrality of the canal between the groups for the studied points. * indicates .05 level of significance ** indicates .01 level of significance

Sections	Preparation system		P-value
Section A	Reciproc Blue	WaveOne Gold	0.77
		AF Blue R3	0.003**
	WaveOne Gold	AF Blue R3	0.007**
Section B	Reciproc Blue	WaveOne Gold	0.162
		AF Blue R3	0.000**
	WaveOne Gold	AF Blue R3	0.001**
Section C	Reciproc Blue	WaveOne Gold	0.274
		AF Blue R3	0.000**
	WaveOne Gold	AF Blue R3	0.006**
Section F	Reciproc Blue	WaveOne Gold	0.007**
		AF Blue R3	0.061
	WaveOne Gold	AF Blue R3	0.38
Section G	Reciproc Blue	WaveOne Gold	0.01**
		AF Blue R3	0.041*
	WaveOne Gold	AF Blue R3	0.554

The mean values of the canal centrality preservation rate were also calculated at three thirds (coronal, middle, and apical) among the three groups (Table 4).

**Table 4:** The arithmetic mean and standard deviation of the values of the canal centralization rate at the three third (coronal, middle, and apical).

Sections	Preparation system	Number of measures	Mean	Standard deviation	Min	Max
Coronal third	Reciproc Blue	15	0.781	0.087	0.643	0.963
	WaveOne Gold	15	0.746	0.107	0.545	0.952
	AF Blue R3	15	0.586	0.085	0.421	0.736
Middle third	Reciproc Blue	15	0.469	0.196	0.168	0.793
	WaveOne Gold	15	0.637	0.206	0.224	0.919
	AF Blue R3	15	0.598	0.175	0.281	0.950
Apical third	Reciproc Blue	15	0.497	0.132	0.322	0.696
	WaveOne Gold	15	0.531	0.151	0.271	0.810
	AF Blue R3	15	0.517	0.136	0.310	0.769
Entire canal	Reciproc Blue	15	0.576	0.084	0.422	0.761
	WaveOne Gold	15	0.630	0.114	0.427	0.806
	AF Blue R3	15	0.563	0.095	0.436	0.787

To study the effect of the three studied systems on the rate of maintaining centrality, the one-way ANOVA test was applied (Table 5). The results show that there were no statistically significant differences in the rate of maintaining the centrality of the canal in the middle third, apical third, and entire canal.

**Table 5:** The results of one-way ANOVA tests for the three third (coronal, middle, and apical). * indicates .05 level of significance ** indicates .01 level of significance

Variable	Sections	ANOVA results	p-value
Canal centralization	Coronal third	18.581	0.000**
	Middle third	3.11	0.055
	Apical third	0.215	0.807
	Entire canal	1.94	0.156

On the other hand, there were statistically significant differences in the rate of maintaining the centrality of the canal in the coronal third (p-value = 0.000). For two-group comparisons, the Bonferroni test was applied (Table 6). 

**Table 6:** Bonferroni test results for comparison, maintaining the centrality of the canal between the groups in the coronal third. * indicates .05 level of significance ** indicates .01 level of significance

Preparation system		p-value
Reciproc Blue	WaveOne Gold	0.933
	AF Blue R3	0.000**
WaveOne Gold	AF Blue R3	0.000**

The results show in Table 6 that there was a statistically significant difference in the rate of maintaining the centrality of the canal in the coronal third, where the Reciproc Blue and WaveOne Gold groups outperformed the AF Blue R3 group.

A one-way ANOVA test was conducted to study the significance of the differences in the mean preparation time (Table 7). For two-group comparisons of preparation time, the Bonferroni test was performed. It was found that there were statistically significant differences in the time of canal preparation between the three groups, as the canal preparation in the WaveOne Gold group took the least time (121.47 seconds), followed by the Reciproc Blue group (192.47 seconds) and the AF Blue R3 group (287.87 seconds) (Table 8).

**Table 7:** One-way ANOVA results for comparison preparation time between the groups. * indicates .05 level of significance ** indicates .01 level of significance

Time	Mean	Standard deviation	F	p-value
Reciproc Blue	192.4667	29.54142	179.7	0.000**
WaveOne Gold	121.4667	4.5492		
AF Blue R3	287.8667	29.19361		
Total	200.6	72.8627		

 

**Table 8:** Bonferroni test results for comparison preparation time between the groups. * indicates .05 level of significance ** indicates .01 level of significance

Group (1)	Group (2)	Mean difference (1-2)	Standard Error	p-value
Reciproc Blue	WaveOne Gold	71	8.80822	.000**
	AF Blue R3	-95.4	8.80822	.000**
WaveOne Gold	AF Blue R3	-166.4	8.80822	.000**

## Discussion

The introduction of nickel-titanium constituted an important development in the endodontic field due to its superiority in preserving the original anatomical shape of the root canal with its high flexibility. This pushed manufacturers to develop the best file mixtures and designs to allow for the best cleaning of various anatomical shapes of the canal system while keeping this preparation as gentle as possible to preserve the greatest amount of dentinal structure [9].

Several studies have been conducted to analyze the properties of rotary files, such as cutting efficacy during preparation, preservation of root canal anatomical path, and preparation time [10]. Several nickel-titanium files with various alloys have been developed in an attempt to transition from multi-file preparation systems to single-file preparation systems to reduce working time and increase clinical efficiency [11]. Therefore, this study aimed to evaluate the effectiveness of three rotary single-file and reciprocating systems in the preparation of highly curved simulated canals to study the changes that will occur in the centrality of the original canal.

The AF Blue R3 system is manufactured using nickel-titanium alloy (AFTM-wire) that is heat-treated with special technology. This system is characterized by good flexibility, strong mechanical properties, and sufficient cutting strength [12]. When studying the centrality of the canal, the results of this study showed there were no differences between the Reciproc Blue and WaveOne Gold systems. This is due to the design of the WaveOne Gold file with an alternating parallelogram cross-section and four cutting edges, but only two edges connect with the canal every 200 microns, which keeps the file centered within the longitudinal axis of the root [13].

The good ability of the Reciproc Blue system to keep the canal centralized can also be explained by the design of the file as an s-shaped spiral with two cutting edges with a non-working head, and a variable taper along the file. This design ensures that the stress is distributed over all the curvature parts of the canal and thus allows for a uniform cut in the curvature area [14].

For the coronal third, this study showed the superiority of the Reciproc Blue and WaveOne Gold systems compared to the AF Blue R3 system. This can be explained by the rectangular cross-section of the AF Blue R3 file with a constant taper rate of 6% along the length of the file and the AFTM-Wire's mechanical properties, including its strength and high rigidity, which allow for increased cutting efficiency of the file.

The results of this study showed that there were no statistically significant differences in the entire canal. However, the WaveOne Gold system showed better values in maintaining the centrality of the canal, followed by the Reciproc Blue system and then the AF Blue R3 system. This can be attributed to the small diameter of the WaveOne Gold file primary (0.7-25), where the WaveOne Gold file has a variable taper of 0.7 of D0-D3, 0.6 of D4-D8, and 0.3 of D9-D16 compared to the Reciproc Blue file (0.8-25), which has a variable taper of 0.8 of D0-D3, and 0.4 of D4-D16. While the AF Blue R3 file has a constant taper along the length of the file, the flexibility of the mixture may have the greatest impact on the performance of the file.

This study agreed with the study by Elashiri et al., where they noted that there were no differences between WaveOne Gold and Reciproc Blue systems [15]. This study also agreed with Zahid et al., where it was noted that the WaveOne Gold system shows a high concentrating capacity with lower canal transfer compared to the Reciproc Blue system [16]. Elashiri et al. also noted that there were no differences between WaveOne Gold and Reciprocal Blue systems in the canal deviation in the coronal and middle sections and the superiority of WaveOne Gold in the apical section, and this is consistent with the results of the current study [15].

This study differed from Silva et al., who found no statistically significant differences between the preparation systems (Reciproc Blue and WaveOne Gold), despite Reciproc Blue's superiority in maintaining centrality and apical foramen transmission, which was followed by the WaveOne Gold cooler. This difference can be attributed to the different imaging methods and the number of levels used to study the centrality of the canal in this study [17].

The WaveOne Gold system required a shorter preparation time compared to Reciproc Blue and AF Blue R3. This can be attributed to the high cutting properties of the WaveOne Gold file and the design of the file in an alternating parallelogram with two cutting edges at an angle of 8.5° over the entire working length. This study agreed with the study by Kataia et al., where they found that WaveOne Gold had a lower preparation time for the canal when compared to Reciproc Blue [18].

Limitations

This study was done on simulated root canals, and this is the major limitation of the study. This is because the cutting efficacy can differ between dentin and resin.

## Conclusions

It can be concluded that the systems of both Reciproc Blue and WaveOne Gold maintained anatomical shape and canal centrality, with WaveOne Gold being more advanced. Regarding the volumetric changes, AF Blue R3 had the greatest changes compared to the Reciproc Blue and WaveOne Gold systems. Results of the canal preparation time showed the Wave One gold group took the least time among the three investigated groups. Future studies are required to investigate the efficiency of different systems in the instrumentation of curved root canals.
